# Frailty and genetics: linking molecular aging to clinical vulnerability

**DOI:** 10.1093/gerona/glag088

**Published:** 2026-04-11

**Authors:** Jhuliana Castillo, Natalia García, Fernando Gómez

**Affiliations:** Research Group on Geriatrics and Gerontology, Faculty of Health Sciences, Universidad de Caldas, Manizales, Colombia; Head of the Research Office, Vicerrectoría de investigaciones, Universidad de Caldas, Manizales, Colombia; Research Group on Geriatrics and Gerontology, Faculty of Health Sciences, Universidad de Caldas, Manizales, Colombia; (Biological Sciences Section)

**Keywords:** Aging, Molecular pathways, Single-nucleotide polymorphisms, Inflammaging, Inflammatory cytokines

## Abstract

Aging is a multifactorial process involving cumulative cellular and molecular damage, directly affecting physiological reserve and homeostasis. In this context, frailty emerges as a complex, dynamic, and potentially reversible geriatric syndrome, associated with inflammatory dysregulation, immunosenescence, and genetic alterations. This narrative review presents the most recent findings linking frailty to genetic factors, including genome-wide association studies and specific polymorphisms related to inflammation. The genetic relationship between frailty and various chronic comorbidities is also explored. This genetic perspective provides a promising framework for a better understanding of the etiopathogenesis of frailty and highlights new opportunities for individualized interventions.

## Introduction

Aging results from the accumulation of molecular and cellular damage over the lifespan, driven by a decline in the regulation of maintenance and repair networks.^1^ The precise threshold of damage required to produce significant alterations in physiological function remains uncertain. It is from these varying levels of damage that physiological reserve emerges, which is essential for compensating age- and disease-related changes.^2^

Biological hallmarks of aging include genomic instability, loss of proteostasis, telomere attrition, epigenetic alterations, dysregulated nutrient sensing, mitochondrial dysfunction, cellular senescence, altered intercellular communication, and stem cell depletion. These processes result in impaired homeostasis and compromised stress responses, both of which are closely linked to physiological reserve.^3^

As a result of these age-related changes, older adults become more susceptible to developing cardiovascular disease, dementia, stroke, diabetes mellitus, and cancer.^3^ As previously mentioned, these molecular and genetic alterations affect homeostasis and its interaction with various biological and environmental factors, potentially leading to the development of frailty.^4^ Frailty is a physiological state characterized by the dysregulation of multiple systems in the aging organism, resulting in reduced homeostatic capacity that increases vulnerability to disability, disease, and ultimately death. It should be noted, however, that frailty is not synonymous with comorbidity or disability; rather, comorbidity is an etiological risk factor, and disability is a consequence of frailty.^4^^,^^5^

## Frailty: Definition and Epidemiology

The prevalence of frailty increases progressively with age. A global analysis reports that among individuals aged 50-59, prevalence is approximately 11%, whereas in those over 90 years it can exceed 50%.^6^

Frailty is a complex phenotype of aging that increases the risk of disability and mortality.^3^ For this reason, in recent years, geriatrics and gerontology have increasingly focused on its study. Multiple definitions of frailty have been proposed. Some consider it as an accumulation of deficits measured by a frailty index (FI), while others define it as a clinical syndrome closely associated with loss of homeostasis.^4^

However, the most commonly used frailty criteria are those defined by Linda Fried: unintentional weight loss in the past year, self-reported exhaustion, slow walking speed, low levels of physical activity, and reduced grip strength.^5^

Thus, frailty is not a disease but a geriatric syndrome that identifies individuals at higher risk of adverse health outcomes. It is important to note that frailty is a “dynamic condition,” meaning that not all individuals present frailty in the same way. Moreover, a frail individual can revert to a non-frail state if frailty is identified and addressed promptly.^7^

Globally, the prevalence of frailty among community-dwelling older adults varies according to the diagnostic method employed. Studies based on the physical phenotype (eg, Fried’s criteria) report a global prevalence of approximately 12% (95% CI: 11%-13%), whereas those using the deficit accumulation model (FI) report figures around 24% (95% CI: 22%-26%) in individuals aged 50 years and older. Pre-frailty is even more common, with prevalence estimates ranging from 46% to 49%, depending on the diagnostic method employed.^6^^,^^8^

Women exhibit higher rates of frailty than men, both in studies based on the physical phenotype (15% vs 11%) as in the FI (29% vs 20%).^8^^,^^9^ In addition, frailty is more prevalent among institutionalized individuals (eg, hospitals and nursing homes), in low- and middle-income countries, and among those experiencing social vulnerability.^6^^,^^10^

## Frailty and Aging

When studying the pathophysiology of frailty, rather than identifying a single sequential pathway, a substantial role of inflammation has been described. Sustained low-grade inflammatory responses, even in the absence of an active trigger, can persist over time and generate a state of chronic inflammation, which has emerged as a key contributor to frailty.^2^ In recent years, age-related changes in the immune system have also been investigated, particularly intrinsic defects in T cell responsiveness, using models in older adult mice.^11^ Additionally, alterations in CD4+ lymphocytes have been observed, including disruptions in signaling pathways.^11^^,^^12^ These age-related alterations in T-cell signal transduction may represent a key contributor to immune dysfunction, being partly responsible for the persistent low-grade inflammatory state observed in older adults.^13^

Additionally, genetic factors have been investigated within the study of this clinical and dynamic syndrome. Their role has been controversial, raising questions about the influence of individual genetic backgrounds on frailty.^14^

Epidemiological risk factors for frailty include advanced age, female sex, low socioeconomic status, the presence of chronic comorbidities (particularly depression and malnutrition), disability in activities of daily living, and negative self-perception of health.^9^^,^^10^ In rural areas, the prevalence of frailty may be higher due to limited access to healthcare resources and greater social vulnerability.^10^

In response to the ongoing etiological investigation of frailty, a bifactorial model has been proposed, comprising a general factor representing genetic overlap across all deficits and 6 residual factors reflecting shared genetic signals within specific groups of deficits: limited social support, unhealthy lifestyle, multimorbidity, metabolic disorders, impaired cognition, and disability. This approach has prompted the use of statistical methodologies that address the limitations of traditional definitions, which can obscure certain etiological pathways. Consequently, genomic structural equation modeling (genomic SEM) has emerged as a recent tool for analyzing the genetic basis of frailty at a multidimensional level, overcoming the constraints of aggregated measures.^15^

The objective of this narrative review is to present the most relevant findings on the relationship between genetics and frailty, focusing on twin studies, specific polymorphisms, and genome-wide association studies (GWAS).

## Heritability of Frailty: Evidence from Twin Studies

An example of the genetic basis of frailty can be seen in findings from twin studies conducted in the United Kingdom, Sweden, and Denmark.^4^^,^^16^^,^^17^ The first of these studies, conducted in Denmark and published in 2012, used a distinct twin design in which participants had been raised in different environments. This approach allowed the researchers to estimate the heritability of frailty, showing that approximately 43% of the variability in frailty could be attributed to genetic factors. These findings suggest that genetic influences may play an important role in the development of this phenotype.^4^

Subsequently, a study conducted in the United Kingdom demonstrated that frailty is influenced by both genetic and environmental factors in twins, with 45% of the interindividual variation in the FI attributable to additive genetic effects and 52% to the individual’s unique environment. This cross-sectional analysis also found that childhood socioeconomic status and several health-related behaviors—including education, marital status, physical activity, smoking, and alcohol consumption—significantly influenced frailty ^17^. Similarly, a longitudinal study conducted in Sweden examined how these factors evolve over time, showing that frailty (measured using the FI) increases 4- to 5-fold more rapidly after the age of 75. Although the heritability of frailty at age 75 remained moderate (42% in men and 55% in women), consistent with cross-sectional findings, the increasing variability of frailty in advanced age was mainly attributed to individual-specific environmental influences rather than genetic factors (Table 1).^16^

**Table 1:** Twin studies and heritability estimates of frailty.

Author	Year	Country	Study type	Heritability estimate	Reference
**Dato et al.**	2012	Denmark	Longitudinal	43% (cluster analysis)	^4^
**Young et al.**	2016	United Kingdom	Transversal	19% (Fried phenotype)	^17^
**Mak et al.**	2023	Sweden	Longitudinal	30%-45% (FI)	^16^

Source: Own elaboration based on references 4, 17, and 16. Fragility Index (FI).

It should be noted that within a population of relative genotypic homogeneity, subtle single nucleotide changes in the DNA sequence—such as single-nucleotide polymorphisms (SNPs) or rare variants—may occur in gene coding sequences (exome), non-coding regions, or intergenic regions, potentially explaining the predisposition to develop a specific phenotype.^14^

Twin studies have provided an important framework for estimating the relative contribution of these genetic factors to frailty. However, variability in heritability estimates across studies may partly reflect differences in study design and population characteristics. For example, some studies have relied on specific national twin cohorts or smaller samples of older adults, while others have analyzed larger longitudinal datasets covering broader age ranges and frailty trajectories.^4^^,^^16^^,^^17^

## Chronic Inflammation, Immunosenescence, and Genetic Polymorphisms in Frailty

Establishing whether frailty is related to genetic characteristics is a relatively recent research goal. Low-grade chronic inflammation is a central aspect of these studies. Age-related changes in the immune system lead to a persistent low-grade systemic inflammatory state, termed “inflammaging.” This phenomenon is characterized by elevated levels of inflammatory mediators and is associated with increased morbidity and mortality in older adults, significantly contributing to frailty.^18^

A significant increase in serum levels of interleukin-6 (IL-6), C-reactive protein (CRP), and tumor necrosis factor-α (TNF-α) has been documented in frail and pre-frail individuals. For instance, elevated IL-6 levels predict an increased risk of physical disability and reduced muscle strength.^18^

Several studies have attempted to link inflammatory molecules, such as interleukins and CRP, with frailty. A 2016 meta-analysis including 32 cross-sectional studies and 3 longitudinal studies found that, in the cross-sectional studies, serum IL-6 and CRP were associated with frailty, whereas the 3 longitudinal studies indicated that these inflammatory markers were not predictors of incident frailty.^19^ However, these measurements were not analyzed from a genetic perspective. In contrast, the 2016 Longitudinal Aging Study identified that the promoter variant rs1800629 in the pro-inflammatory cytokine TNF gene was associated with frailty using the syndromic definition, and in the same cohort, rs360722 in the IL18 gene and SNPs 34DX2X (rs4679868 and rs9852519) in the IL12 gene were associated with frailty using the cumulative deficit score.^20^

Similarly, in the search for SNPs in genes related to inflammation, several studies have reported associations with frailty. Almeida et al. identified a relationship between the rs360722 promoter variant of the CRP gene and frailty.^21^ Later, Cedillo et al. and Mourtzi et al. described associations with rs180087 and rs1800896 in the IL10 gene, and rs429358 and rs7412 in the APOE gene, respectively (Table 2).^22^^,^^23^

**Table 2:** Key genes associated with frailty and their functional processes.

Gene	Dominant macro-categories	Representative terms (GO/Reactome)	Interpretation in frailty
**APOE**	Metabolism/Energy; Neuroplasticity/Cognition	Lipid metabolism; cholesterol transport; Alzheimer’s disease	Risk of cognitive and metabolic frailty due to lipid dysregulation.
**TNF**	Inflammation/Cytokines; Apoptosis/Stress	Inflammatory response; TNF signaling; apoptotic process	Inflammatory hub; supportive of inflammaging and sarcopenia
**SMAD3**	TGF-β/Remodeling; Apoptosis/Stress	TGF-β signaling; extracellular matrix organization	Tissue remodeling/fibrosis; regulation of aging
**CD4**	Adaptive immunity/MHC; Inflammation/Cytokines	T cell activation; adaptive immune response	Adaptive immunosenescence; loss of responsiveness
**PAFAH1B1**	Metabolism/Energy; Neuroplasticity/Cognition	Brain development; axon guidance; neuronal migration	Neurodevelopmental disorders; link with cognitive frailty
**IL10**	Inflammation/Cytokines; Adaptive immunity	Cytokine-mediated signaling; negative regulation of immune response	Dual immunomodulatory role; immune-inflammatory frailty
**HLA-DRB1**	Acquired immunity	Antigen processing; MHC Class II protein complex	Alteration of antigenic presentation; increased infectious risk
**HTT**	Neuroplasticity/Cognition; Adhesion/Migration	Synapse organization; neuron projection	Cognitive and motor fragility; neurodegeneration
**CRP**	Inflammation/Cytokines; Apoptosis/Stress	Acute-phase response; response to stress	Clinical biomarker of systemic frailty
**IL12B**	Inflammation/Cytokines; Adaptive immunity	Interleukin-12 signaling; T cell differentiation	Link between chronic inflammation and adaptive response

Source: Own elaboration.

However, the strength and consistency of these associations vary across studies. This heterogeneity may be explained by differences in the inflammatory pathways and genetic variants analyzed, as well as by variations in study populations, sample sizes, and operational definitions of frailty. In addition, methodological differences between candidate gene approaches and broader genomic analyses may further contribute to the variability in reported findings.

## Genetic Architecture of Frailty: Evidence from Genome-Wide Association Studies

Several GWASs investigating the genetic mechanisms underlying frailty have identified approximately 78 susceptibility SNPs across 59 genetic loci, including genes such as NLGN1, SYT14, ARPC5L, and MAP3K3.^24^^,^^25^

Mekli et al. analyzed 3160 individuals aged over 50 years from the English Longitudinal Study of Ageing and reported an association between the FI and GRIN2B.^26^ Atkins et al. conducted a meta-analysis of the FI in European-descent participants from the UK Biobank (*n* = 164 610; aged 60-70 years) and the Swedish TwinGene cohort (*n* = 10 616; aged 41-87 years), identifying 34 loci and estimating SNP-based heritability at approximately 11%.^24^ Similarly, Ye et al. identified 123 loci associated with the frailty phenotype and estimated SNP heritability at about 6%. In contrast, Livshits et al., analyzing 3626 UK female volunteers aged 17-93 years using the FI, found no significant genetic associations.^25^ More recently, Foote et al. performed a multivariate GWAS examining the latent genetic architecture across 30 frailty-related deficits and identified 408 genomic risk loci.^15^ Although the largest of these cohorts approaches the scale commonly observed in GWAS of other chronic diseases of aging, many studies still rely on comparatively smaller samples, which may limit statistical power and the detection of variants with modest effects. Furthermore, most GWAS of frailty have been conducted predominantly in populations of European ancestry, highlighting the need for greater ancestral and ethnic diversity and for analyses in additional large populations to better characterize genetic susceptibility and heritability across different demographic groups.

Estimates from genome-wide association studies suggest that the SNP-based heritability of frailty is lower than the heritability reported in twin studies. For example, GWAS analyses have estimated SNP heritability at approximately 11% for the FI and around 6% for the frailty phenotype,^24^^,^^25^ whereas twin studies have reported heritability estimates ranging from approximately 30% to over 50%. This discrepancy may reflect the contribution of additional genetic factors not captured by common SNPs, including rare variants, gene–gene interactions, and environmental influences across the life course.

## Frailty, genetics, and comorbidities

Growing evidence from genetic and genomic studies suggests that frailty shares genetic architecture with several aging-related diseases. An example is the strong association between chronic widespread pain and frailty, with shared latent genetic factors suggesting a common causal basis rather than pain acting solely as a contributing factor to frailty.^27^ Regarding the relationship with chronic kidney disease (CKD), a Mendelian randomization study provided evidence of a causal association between genetically predicted FI and an increased risk of CKD, even after adjustment for factors such as body mass index and inflammatory markers.^28^ Finally, research examining chronic obstructive pulmonary disease and frailty identified a significant positive genetic correlation (Rg = 0.4324), as well as the presence of approximately 3200 shared causal variants.^29^

Among the specific genes and loci identified as having shared influence in frailty and these diseases are HLA-DRB1, PBX3, SLC22A5/OCTN2, SLMAP, SMAD3, GDF11, ADAM12, GRK4, HTT, HLA-DQA1, HLA-DQB1, and KLC1. These genetic interactions highlight common biological pathways, including telomere maintenance and organic cation transport, suggesting shared mechanisms involved in the development and progression of these conditions.^29^

These findings provide additional context for interpreting GWAS results in frailty, suggesting that part of the genetic architecture of frailty may reflect shared biological pathways with other aging-related conditions.

## Network of gene interactions and frailty

Genes associated with frailty were identified through a narrative review of published studies examining genetic variants linked to frailty phenotypes or frailty-related traits. Studies reporting associations from candidate gene analyses, polymorphism studies, and genome-wide association studies were considered. When the same gene was reported in multiple studies, it was included only once in the final list. This process resulted in a set of 39 genes used for the subsequent network analysis (see supplementary methods).

Based on genes reported in the literature as associated with frailty and related comorbidities—including *TNF, IL18, IL12, IL10, APOE, SYT14, LRPPRC, HTT, HLA complex genes, SMAD3*, and *ADAM12*, among others—a protein–protein interaction network was constructed using Cytoscape (v3.10). The network was generated using the stringApp plugin, with *Homo sapiens* as the reference species, an interaction confidence threshold of 0.70, and no additional interactors added.

Subsequently, network topology analysis was performed using the *Network Analyzer* plugin to calculate centrality measures (degree, betweenness, and closeness). Functional modules were then identified using the MCODE clustering algorithm with the following parameters: degree cutoff ≥ 2, node score cutoff 0.2, and k-core ≥ 2.

**Figure 1:**
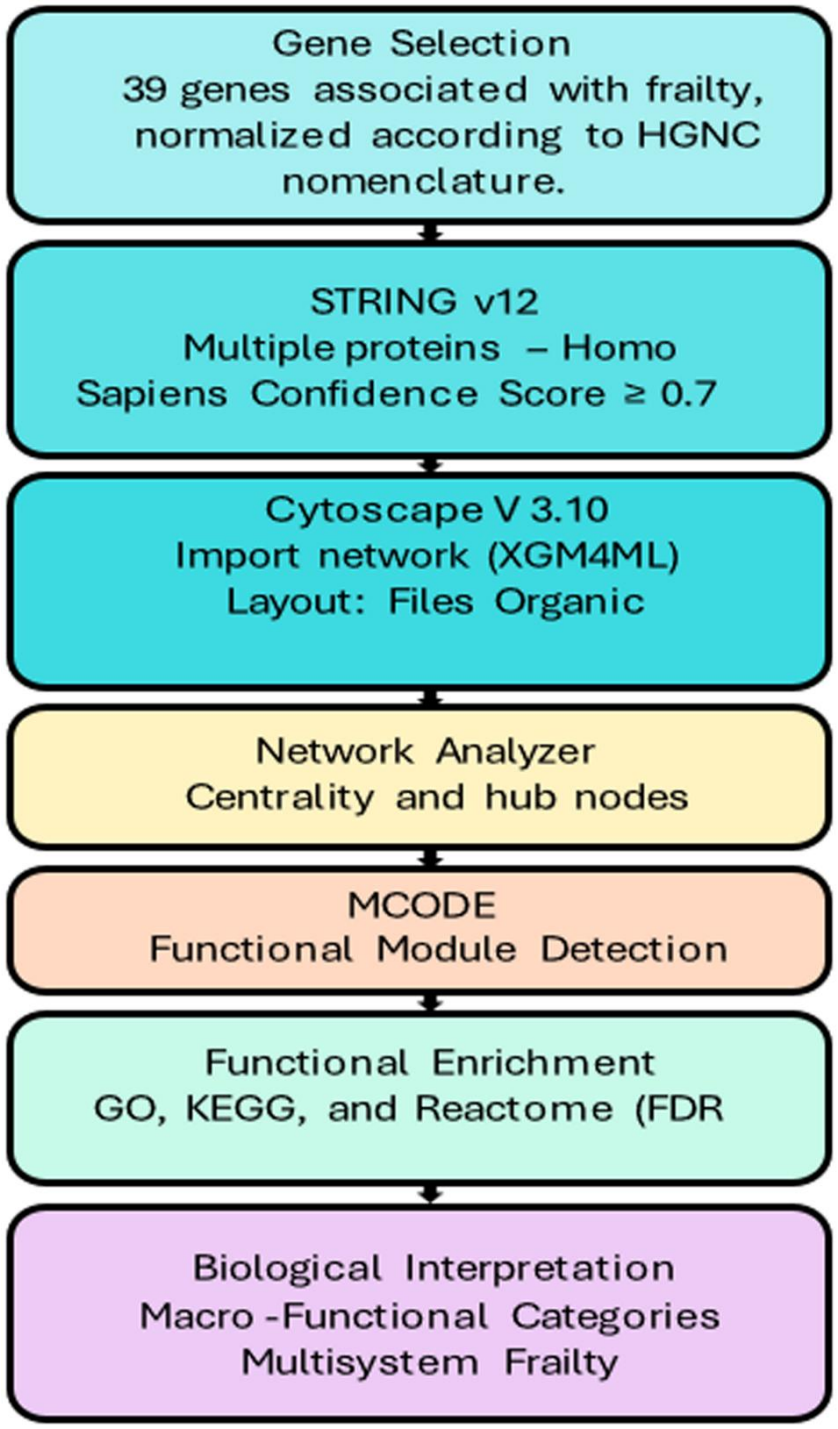
Methodological workflow of gene networks and frailty. Source: Own elaboration.

Functional enrichment analysis was performed using Reactome FIViz and stringApp, exploring Gene Ontology categories (GO: Biological Process) as well as KEGG and Reactome pathway databases. Associations with an adjusted false discovery rate ≤ 0.05, corrected using the Benjamini–Hochberg method, and involving at least 3 contributing genes per term were considered significant (Figure 1).

Finally, the results were integrated and visualized using EnrichmentMap and AutoAnnotate, generating clusters of biological pathways and semantic annotations that grouped the findings into modules related to inflammation, energy metabolism, neuroplasticity, and immune signaling. The networks and enrichment tables were exported in vector formats (SVG/PDF) and as CSV files to ensure reproducibility.

This visual organization should be interpreted as a representation of network topology within the analyzed dataset rather than as a direct ranking of overall biological importance across all frailty-related pathways.

**Figure 2:**
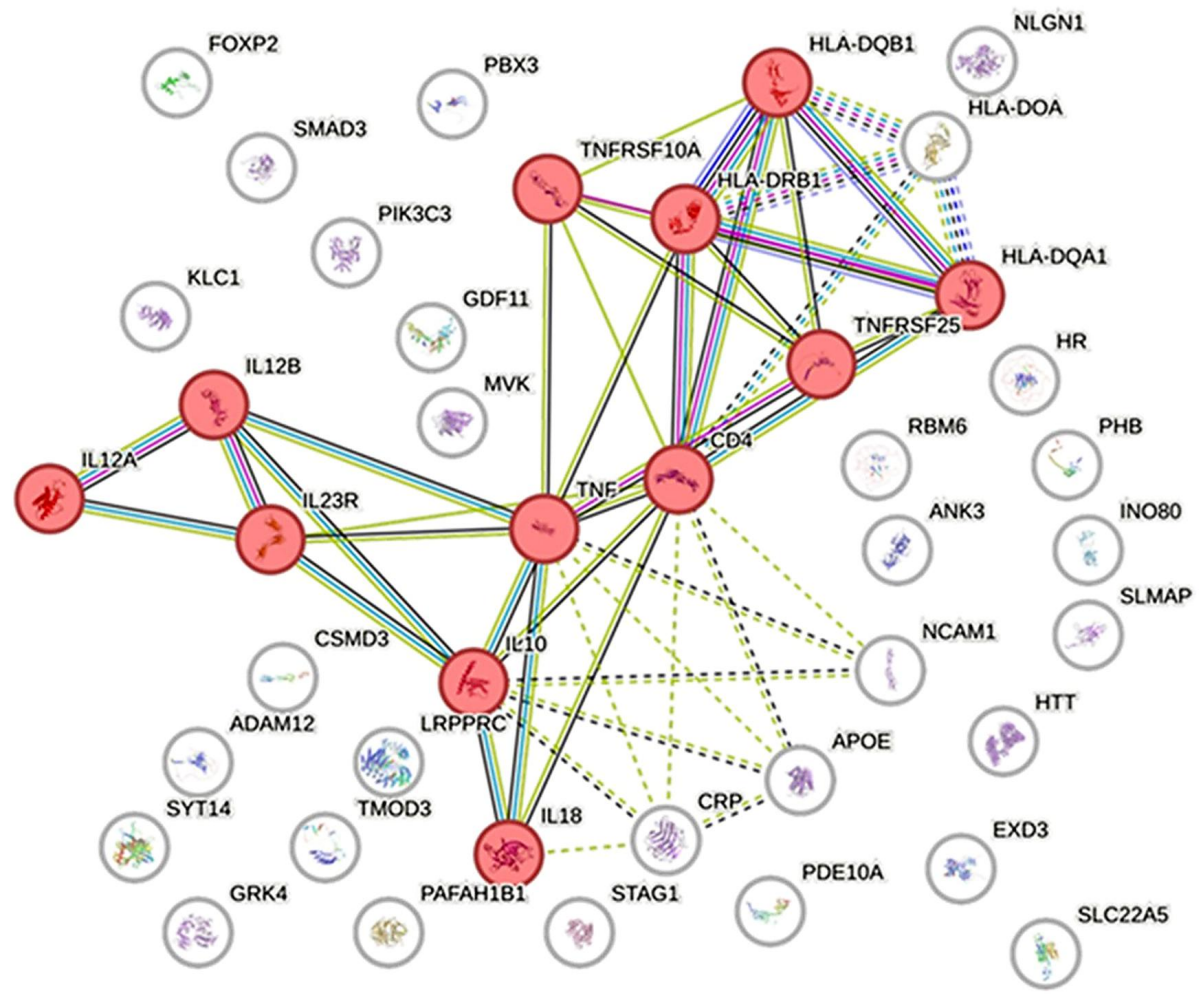
Gene network and frailty. Source: Own elaboration. Interaction landscape of genes associated with frailty, represented as a network generated in Cytoscape for academic and interpretative purposes. Each node corresponds to a gene, and each edge represents a documented interaction between gene products according to the selected network source and interaction settings. Node colors were used to distinguish the principal genes integrated into the core interaction module from secondary or less-connected associated genes. Node size reflects the relative topological relevance of each gene within the network, as inferred from its connectivity pattern in the displayed graph. Edge colors represent distinct interaction types included in the network construction. The figure highlights a central, highly interconnected module enriched in inflammatory and immune-related mediators, whereas several genes remain peripheral or disconnected, indicating lower integration into the principal interaction structure.

The network indicates that the greatest functional convergence is concentrated in the central interconnected genes of the main module, with TNF occupying a prominent position within a core set of genes related to inflammatory and immune-associated signaling. Together, these genes define the most topologically integrated and biologically coherent sector of the interaction map, consistent with a shared role in the molecular processes underlying frailty (Figure 2).

Although APOE is positioned as a peripheral node in the present network, it remains biologically relevant due to its well-established role in lipid metabolism and its association with cognitive impairment. The APOE gene is widely recognized for its association with Alzheimer’s disease; however, increasing evidence also links APOE variants with frailty through mechanisms involving lipid metabolism, neuroinflammation, and cognitive decline, which are key components of the multidimensional frailty phenotype. This supports the concept of frailty as a neurocognitive syndrome, even if APOE does not occupy a central topological position in this specific interaction map.^23^^,^^24^ These observations highlight the distinction between topological centrality within the constructed network and broader biological relevance derived from prior evidence. TNF and IL10 define the core of the inflammatory axis, which is closely associated with sarcopenia and functional decline linked to chronic inflammation. Immune-related genes such as CD4 and IL12B were also included due to their roles in T-cell activation and pro-inflammatory cytokine signaling. These pathways contribute to chronic low-grade inflammation and immunosenescence, biological processes strongly implicated in the development of frailty.^19^^,^^20^ The HLA-DRB1, HLA-DQA1, and HLA-DQB1 genes cluster within antigen presentation pathways and are implicated in the immune dysregulation associated with immunosenescence in advanced age. Conversely, genes associated with neuroplasticity, such as HTT, NCAM1, and PAFAH1B1, highlight the convergence between physical and cognitive components of frailty. These findings reinforce the concept of frailty as a convergent multisystem phenotype. Within the complex network of biological interactions underpinning clinical vulnerability (Table 2), inflammation emerges as the most topologically integrated process, while metabolic and neurocognitive pathways contribute complementary biological dimensions supported by prior evidence.

## Current and future perspectives in addressing frailty: from clinical to genomic approaches

Due to the increase in life expectancy and the decline in fertility rates in recent decades, the proportion of people over 60 years old is rapidly increasing, leading to a progressive inversion of the population pyramid.^30^^,^^31^

This increase in the older adult population has generated significant interest in the study of aging and in addressing the problems associated with it.^1^^,^^32^ Traditionally, both clinical practice and medical research have focused on survival and interventions aimed at increasing longevity. However, with the emergence of the concept of healthy aging, the current trend emphasizes the prevention of disability over the mere extension of lifespan.^33^

This scenario highlights once again that frailty is a “dynamic condition,” potentially reversible if identified and treated in a timely manner.^14^ Older adults can live for years in this state before death, often experiencing a progressive decline in their quality of life.^34^ Therefore, addressing this geriatric syndrome requires multidimensional approaches and perspectives—biological, cellular, and genetic, among others.

The genetic perspective has gained great relevance, particularly with the identification of variants in coding regions of the genome (exomic variants). These findings not only help assess the risk of developing specific pathologies but also provide valuable tools for influencing prognosis and treatment response.^14^

Regarding the management of frailty, several clinical interventions are currently available, including physical activity, protein-calorie supplementation, and the withdrawal of unnecessary medications. However, the effectiveness of these strategies is not yet supported by a strong evidence base.^35^ In relation to physical activity, it has been reported that genetic variations in pathways involved in frailty—such as those related to the immune system, apoptosis, and autophagy—significantly influence both tolerance to and adherence with exercise interventions.^36^

This suggests that the genomic model proposed by Foote et al. can refine patient risk stratification and facilitate the development of more targeted preventive and therapeutic strategies that address the specific biological mechanisms underlying each subcomponent of frailty.^15^

Building on this perspective, the growing integration of genetic data into frailty research opens the possibility of advancing toward more individualized strategies for prevention and management, in line with the principles of precision medicine. Genomic approaches may contribute to earlier identification of individuals at higher risk through polygenic prediction and causal inference analyses. In addition, the characterization of frailty-related genetic traits could help guide therapeutic decision-making and evaluate the effectiveness and safety of targeted interventions. Identifying biological heterogeneity among subgroups of older adults with frailty may also improve the accuracy of clinical management strategies. Finally, at the population level, genomic research may support the identification of susceptibility to risk factors and inform preventive strategies.

## Conclusions

Frailty represents a central challenge in population aging, carrying significant clinical, functional, and social implications. While the phenotypic characterization of frailty has advanced considerably, understanding its molecular and genetic bases remains limited. Current evidence suggests the existence of relevant genetic determinants—including specific polymorphisms and variants identified through genome-wide association studies—that may influence both susceptibility to frailty and response to interventions. This emerging genetic dimension opens the possibility of advancing toward more personalized strategies for the prevention and management of frailty, in line with the principles of precision medicine. Further large-scale GWAS will be necessary to more thoroughly characterize the genetic determinants of frailty and the biological pathways involved, potentially providing new insights for interventions aimed at preventing or delaying frailty and promoting healthier and more independent aging. Consolidating knowledge in this field will require robust longitudinal studies, multi-omics approaches, and effective integration between geriatric clinical practice and genomic research.

## Supplementary Material

glag088_Supplementary_Data

## Data Availability

All data and materials analyzed in this study are included within this manuscript and its supplementary materials.
