# Supplementary material for: Frailty and genetics: linking molecular aging to clinical vulnerability
Source: J Gerontol A Biol Sci Med Sci. 2026 Apr 11;81(5):glag088. doi: 10.1093/gerona/glag088 (PMC13120882; doi:10.1093/gerona/glag088)
Supplement: glag088_Supplementary_Data [file glag088_supplementary_data.pdf]

Frailty and Genetics: Linking Molecular Aging to Clinical Vulnerability

Supplementary Methods: Gene Selection, Protein–Protein Interaction Network, and Functional Enrichment Analysis

Gene selection
The analysis started from a curated list of 39 genes previously reported in the literature as being associated with frailty, chronic inflammation, immunosenescence, energy metabolism, and neurocognitive processes. Gene symbols were standardized according to the nomenclature approved by the HUGO Gene Nomenclature Committee (HGNC) to ensure consistency and traceability.

Construction of the protein–protein interaction network
Functional interactions among the selected genes were explored using the STRING v12.0 database (https://string-db.org), applying the *Multiple proteins* option. *Homo sapiens* (taxon ID 9606) was used as the reference organism.
The following parameters were applied:
• Minimum interaction score (confidence score): 0.700, corresponding to high-confidence interactions.
• Maximum number of additional interactors: 0, so that only the connections among the genes of interest were represented.

The resulting network was exported in XGMML format and imported into Cytoscape v3.10 (https://cytoscape.org) for analysis and visualization. The yFiles Organic layout was applied to organize the network and facilitate the identification of functional modules.

Topological analysis
The structural characterization of the network was performed using Cytoscape's *Network Analyzer* tool, which allowed calculation of centrality metrics, including degree and betweenness centrality. This made it possible to identify hub nodes, defined as genes with the highest number of connections and therefore with a potential integrative role in frailty-related processes.

Module detection
To identify densely connected subnetworks, the MCODE (Molecular Complex Detection) algorithm integrated into Cytoscape was used. The parameters were set as follows: degree cutoff = 2, k-core = 2, node score cutoff = 0.2. The resulting modules were interpreted as potential clusters of genes involved in shared biological processes.

Functional enrichment analysis
Functional enrichment was assessed in STRING based on Gene Ontology annotations (GO: Biological Process, Molecular Function, Cellular Component), Reactome Pathways, and KEGG Pathways, applying the Benjamini–Hochberg correction to control for type I error. Terms with FDR $\leq$ 0.05 and at least three implicated genes were considered significant.

To validate the findings, the gene list was also analyzed using g:Profiler (https://biit.cs.ut.ee/gprofiler), including Reactome, KEGG, and GO gene sets. Results from both platforms were compared in order to identify processes robustly associated with frailty.
